# The Use of the Intensive Longitudinal Methods to Study Financial Well-Being: A Scoping Review and Future Research Agenda

**DOI:** 10.1007/s10902-021-00381-6

**Published:** 2021-04-02

**Authors:** Angela Sorgente, Casey J. Totenhagen, Margherita Lanz

**Affiliations:** 1 Department of Psychology, Università Cattolica del Sacro Cuore, Milan, Italy; 2 Department of Human Development and Family Studies, University of Alabama, Tuscaloosa, AL USA

**Keywords:** Financial well-being, Financial stress, Intensive longitudinal methods, Daily diary, Scoping review

## Abstract

Financial well-being is a positive financial condition that has an objective (e.g., income) and a subjective (e.g., financial satisfaction) side. Much research has examined financial well-being using cross-sectional and classic longitudinal designs. More recently, researchers have begun to examine financial well-being using intensive longitudinal designs, collecting data in a repeated (at least five measurements) and intensive (short time interval between measurements) way. The goal of the current study was to systematically review all published research on financial well-being using intensive longitudinal methods, summarize themes from this work, and suggest future research directions. Searching three databases (Scopus, PsycINFO, Econpapers), we found nine articles that respected inclusion and exclusion criteria. From each selected article, we extracted information about (1) research field diffusion, (2) data collection methods, (3) financial well-being’s definition and operationalization, (4) research questions addressed and (5) data analysis. Findings showed that most of the studies adopted an interval-contingent research design, collecting data once a day; that both the objective and subjective sides of the construct were assessed, and that, most of the time, the construct was conceptualized as financial stress (lack of financial well-being). Different kinds of research questions were addressed across studies and these were often analyzed using multilevel analysis. In the discussion section, future research directions are suggested.

## Introduction

From recently published reviews on financial well-being (Brüggen et al., 2017; Ghazali et al., 2020; Sorgente & Lanz, 2017; Wilmarth, 2020), it is possible to identify different theoretical shifts that have characterized the study of this construct. For example, originally, financial well-being was often considered synonymous with income, under the assumption that those with high income would have greater financial happiness than those with low income (Caputo, 1998; Easterlin et al., 1990). More recently, scholars have suggested this strictly objective measure may not be a complete representation of financial well-being. Financial well-being is also made up of individuals’ *perceptions* of their own financial situation (Brüggen et al., 2017) and financial stressors (Netemeyer et al., 2018). This shift from the objective to the subjective side of financial well-being determined other theoretical consequences, such as some researchers suggesting that the perception of one’s future financial condition could have an effect on present happiness as well (e.g., Iannello et al., 2020; Norvilitis et al., 2003; Sorgente & Lanz, 2019). Early investigations of financial well-being were typically driven by economists (Caputo, 1998; Easterlin et al., 1990), but psychologists began to study the construct in greater numbers with the shifts toward recognizing the subjective and future conditions of financial well-being (Sorgente & Lanz, 2017; Stutzer & Frey, 2012). The theoretical development of the financial well-being literature was also shaped by the 2008 economic recession, which recalling the impact finances have on individuals’ well-being, drove more researchers to investigate the financial domain of life. We can expect that the upcoming crisis due to the worldwide coronavirus pandemic will further increase attention on this life domain.

Results obtained from prior reviews (Brüggen et al., 2017; Ghazali et al., 2020; Sorgente & Lanz, 2017; Wilmarth, 2020) offer an interesting overview of financial well-being. In this paper, we aim to enrich knowledge about financial well-being by focusing specifically on studies that investigated this construct using intensive longitudinal (IL) methods (i.e. “intensive, repeated self-reports that aim to capture events, reflections, moods, pains, or interactions near the time they occur”; Iida et al., 2012, p. 277). IL methods are characterized by many repeated measurements taken over short time intervals (Bolger & Laurenceau, 2013) and are used when the researchers want to investigate a construct that can fluctuate over the course of the same day or across days.

Financial well-being has often been conceptualized as static; indeed none of the previously cited reviews (Brüggen et al., 2017; Ghazali et al., 2020; Sorgente & Lanz, 2017; Wilmarth, 2020) included studies which investigated day-to-day changes in financial well-being. Although scientific interest for the systematic collection of information about daily life started at the beginning of the 1900s (Bevans, 1913; Pember-Reeves, 1913), only recently have statistical and technological supports made this intensive data collection more feasible (Iida et al., 2012). Consequently, research on the study of financial well-being using IL methods is very recent, but suggests that individuals’ perceptions of financial well-being can fluctuate from day-to-day (Totenhagen et al., 2018). For example, perceptions of financial well-being may be higher on days in which individuals receive money (e.g., payday) versus days in which they have to spend money (e.g., paying a loan fee, a bill, etc.). Factors affecting financial well-being at a daily level can be detected only by adopting IL methods, as traditional methods such as cross-sectional or longitudinal methods with longer time intervals between assessments may mask these changes.

Documenting how IL methods have been used to study financial well-being, we aimed to show how they are complementary to classic research methods and provide an opportunity to move the literature forward. IL methods permit nuanced investigation of intra-individual change (*within-person* approach), complementing the body of existing research, which has focused on inter-individual differences (*between-person* approach). We believe that IL methods could be used to help solve open issues in the field of financial well-being. For example, IL methods may be used to help clarify the components of financial well-being and financial stress (see the next section on conceptualizations) as well as how objective (material resources) and subjective (financial satisfaction) aspects of financial well-being are intertwined within persons (vs. between persons). Attention to these issues will have relevant practical implications. Such findings could help identify within-person factors that can affect individuals’ financial behaviors and, in turn, their financial well-being (Bruggen et al., 2017; Shim et al., 2009). In other words, improved understanding of the within-person processes that link objective and subjective experiences of financial well-being, stress, and behavior could help to promote effective practice and policy. Thus, in the current paper, we aimed to systematically review studies that have adopted an IL method for investigating financial well-being to map what has been already done and provide future research directions for researchers seeking to implement these approaches.

### Conceptualizations of Financial Well-Being

Conceptualizations of financial well-being are complex, but key themes from prior research include (1) subjective and objective components, and (2) positive and negative components (i.e., well-being and stress).

#### Subjective and Objective Financial Well-Being

We defined financial well-being using the definition proposed by Sorgente and Lanz (2017) and adopted in different studies (e.g., Cherney et al., 2020; Iannello et al., 2020; Sorgente & Lanz, 2019). These authors define financial well-being as a positive financial condition that has an objective and a subjective side. The former (sometimes referred also as “economic well-being”) consists of the material resources that an individual possesses (e.g., income, assets, saving account, health insurance, job benefits, etc.). Subjective financial well-being consists of an individual’s subjective evaluation with respect to his or her financial condition. This evaluation may include emotional (e.g., feelings of calm or anxiety caused by personal financial experiences of the individual) and cognitive (e.g., the degree of satisfaction one has for his/her financial condition, or “financial satisfaction”) evaluations. Further, subjective evaluations can refer to the present financial condition or to the anticipated future financial condition. Both dimensions are relevant for the individual’s present level of well-being (Iannello et al., 2020).

This definition is the result of the recent growth of literature on financial well-being that has unveiled the complexity of this construct. For example, although conceptualization of financial well-being is usually generalized to the entire population, some studies have shown that it should be tailored according to individuals’ age and culture. Sorgente and Lanz (2017, 2019) found that both objective and subjective financial well-being should be operationalized in a different way when the investigated population is aged 18–29 years. For example, their objective financial well-being should also include economic assistance received from parents, while their subjective financial well-being should include specific components, like the comparisons with peers. Regarding culture, Ghazali et al. (2020) have shown that research about financial well-being developed in western culture could not be confirmed in different cultures. For example, reviewing studies performed on Malaysian citizens, they found that self-esteem was not significantly related to financial well-being (different from what western literature suggests; e.g., Frankham et al., 2020), while culture-specific factors (e.g., Islamic moderating practice) were significantly related to individual financial well-being.

#### Financial Well-Being and Financial Stress

There has been much research on the study of financial well-being in the last decade, yet much remains to be done given the many research gaps that characterize this literature (Brüggen et al., 2017; Sorgente & Lanz, 2017). Among these, a crucial one is the relationship between financial well-being and financial stress. Financial stress has been defined as the non-ability to meet expenses at certain point in time (Kim & Garman, 2003). Financial stress encompasses many attributes of financial circumstances including income, debts, assets, and money management (Osman et al., 2020).

Some scholars believe that financial well-being and financial stress are two sides of the same construct. This is evident both in the way in which they *defined* and *operationalized* financial well-being. For example, Netemeyer et al. (2018) defined financial well-being as a lack of stress related to the management of money. Similarly, many scales measuring financial well-being include reverse-scored items referring to financial stress, such as “I am daily stressed because of my financial situation” (Sorgente & Lanz, 2019) or “I am constantly worried about money” (Shim et al., 2010). On the other hand, others suggest “financial well-being and financial stress are two related but different concepts” (Brüggen et al., 2017; p. 230). This belief is consistent with the distinction between stress (illness) and well-being (wellness) in health sciences. The World Health Organization (WHO) constitution states: “Health is a state of complete physical, mental and social well-being and not merely the absence of disease or infirmity.” Likewise, some scholars conceptualize financial well-being as more than just the absence of financial stress.

As the relationship between financial well-being and financial stress is still a matter of debate (Brzozowski & Visano, 2020), we included in this review studies investigating financial stress to make our review more exhaustive. Like financial well-being, it is also possible to distinguish an objective and a subjective side of financial stress (Sinclair & Cheung, 2016). If the objective side of financial well-being consists of the economic resources that the individual owns (e.g., house) or earns (e.g., income), the objective side of financial stress consists of the economic resources that the individual spends (e.g., expenses, debt; e.g., Hanratty et al., 2007). On the other side, as the subjective dimension of financial well-being consists of a present and future positive view of one’s financial condition (e.g., feeling of satisfaction about one’s financial condition), the subjective dimension of financial stress consists of a present and future negative view of one’s financial condition (e.g., feeling stressed and worried; Heckman et al., 2014). Another aspect that often is taken into consideration is financial stressor events. They are events such as losing a job, receiving an overdue notice from a creditor, getting a phone call from creditor about past due bills, etc. that have the potential to raise the individual’s level of financial stress (Kim et al., 2003). Clearly, financial well-being and financial stress have been defined and operationalized in many ways. Consequently, in our systematic review (see Method section), we adopted different keywords in the syntax referring to financial well-being and stress (e.g., financial satisfaction, financial stress, income, expenses, debts, etc.).

### Intensive Longitudinal (IL) Methods: Research Designs and Advantages

According to Bolger and Laurenceau (2013), an IL study includes enough repeated measurements (at least five) to model a distinct change process for each subject, which can be a person or other sampling unit, such as dyad (e.g., a married couple) or group (e.g., a family). These intensive repeated measurements can happen in different contingencies, and this is the main aspect that determines the classification of IL research designs: interval-, signal-, event-, and device-contingent design (Bolger & Laurenceau, 2013).

In *interval-contingent* designs, participants report their experiences at regular and predetermined intervals of time selected by the researcher. Often, like in daily diary studies, variables are measured prospectively at daily intervals (e.g., reporting each evening how much money was spent during the day), but also studies collecting data more times per day can fit in this research design. Alternatively, the IL design is defined *signal-contingent* when participants report their behavior or experience each time the researcher sends a signal to them. These signals are often provided randomly to obtain a random sampling of real-time thoughts, feelings, and behaviors in context, without involving any retrospection. When the moment in which data are collected is not defined by a signal, but by a predefined event that takes place (e.g., each time the participant spends money), the IL design is labeled *event-contingent*. This design relies on the participant detecting an event and reporting it soon afterwards. Finally, the IL research design is defined *device-contingent* when (at least part of) data collection happens without requiring the participant to use his/her cognitive resources. Most of the data are collected by the device itself. Examples include participants carrying devices (e.g., smartphones) from which the researcher can collect data like physiological indices (e.g., heart rate), environmental indices (e.g., ambient temperature), or spatial data (e.g., GPS information). Each of these four categories of IL designs includes other sub-categories, generating numerous different research designs and consequent labels. According to Bolger and Laurenceau (2013), the most common labels that fit under the IL umbrella are: experience sampling, daily diaries, interaction records, ecological momentary assessment, ambulatory assessment, and real-time data capture.

Despite the specificities of each IL design, the intensive and repeated nature of these methods as a group offer four main advantages over traditional methods. The first advantage is the possibility to reduce the effect of recall biases that often affect traditional measurement. By collecting data repeatedly on the same participants, researchers can answer more detailed research questions, particularly regarding phenomena that change or fluctuate over time (Windt et al., 2018). For example, a question like “did your perception of financial well-being improve the day in which you received your paycheck?” can generate responses that are affected by recall bias. Instead, asking each day “how would you rate your financial well-being today?” generates less biased answered and allows verification of whether the reported level of perceived financial well-being was actually higher the day in which a person received a paycheck as compared to other days. This example introduces the second advantage of IL methods. These research designs allow researchers to directly observe processes of change. In particular, they allow investigation of the pattern of the change (e.g., financial well-being’s fluctuation presents rare but high peaks), the causes of the change (e.g., peaks are due to having received the salary), and the consequences of the change (e.g., in these days the subjects make more expenses).

A third important advantage is that IL methods allow researchers to study behaviors or experiences that are short-lived or otherwise difficult to sample. For example, in the exact moment in which one buys a desired item, one may have a slightly increased heart rate. This change would not be detectable in a traditional cross-sectional study but could be detected in a device-contingent design. Finally, the fourth advantage of IL designs is the possibility to test within-subject processes using within-subject data. Traditional research methods test within-subject hypotheses (e.g., enacting healthier financial behaviors increases perceptions of financial well-being) using between-subject data, verifying for example that people who have healthier financial behavior than others (between-subject comparison) also report higher financial well-being than others. Instead, IL data allow testing of this hypothesis using within-subject data. For example, are the occasions (e.g., days) in which one reports healthier financial behaviors the same occasions in which one reports higher levels of subjective financial well-being? Results obtained using these two approaches could be completely different (Lischetzke, 2014). Importantly, IL methods allow researchers to perform both within- and between-subject comparisons: “intensive longitudinal methods permit us to answer questions within subjects, while also allowing us to determine whether these processes are mirrored in between-subject associations” (Bolger & Laurenceau, 2013, p. 6). In sum, IL methods stand to help move the literature on financial well-being forward by permitting examination of new research questions (e.g., What specific experiences or activities drive immediate changes in subjective financial well-being?), focusing on mechanisms underlying within-person processes and with less recall bias.

### The Current Study

In the current paper, we aimed to introduce IL methods to scholars interested in the study of financial well-being, showing the opportunities that the IL approach can generate in this research field. To do so, we (1) systematically reviewed studies that have already investigated objective or subjective financial well-being adopting an IL approach, in order to show how these studies were performed and what they have discovered, and (2) delineate future research directions for the study of *daily* financial well-being. The systematic review is presented in the methods and results sections, and future directions are presented in the discussion.

## Method

Among the different kinds of knowledge synthesis methodologies (Grant & Booth, 2009; Whittemore et al., 2014), the one that best suits the aims of this study is the scoping methodology. The scoping review “is a form of knowledge synthesis that addresses an exploratory research question aimed at mapping key concepts, types of evidence, and gaps in research related to a defined area or field by systematically searching, selecting, and synthesizing existing knowledge” (Colquhoun et al., 2014, p. 1292).

Our research questions fit within the aims of a scoping review as we were interested in exploring everything that has been done in a specific research area (i.e., the use of IL methods to study financial well-being). We conducted this scoping review following the guidelines originally proposed by Arksey and O’Malley (2005) and later clarified and enhanced by further studies (e.g., Colquhoun et al., 2014; Levac et al., 2010). These guidelines suggest performing the scoping review following five stages (there is also an *optional* sixth stage, which we did not consider adequate to our review’s topic): (1) identifying the research question, (2) searching for relevant studies, (3) selecting studies, (4) charting the data, and (5) collating, summarizing, and reporting the results.

### Identifying the Research Question

The first stage consists of identifying the research questions on which the researchers want to focus. These research questions should be broad in nature as they seek to provide breadth of coverage (Levac et al., 2010). In the current study, we aimed to address the following research questions specific to financial well-being:

1. *Diffusion* In which disciplines is the study of *daily* financial well-being conducted, and where is it more common? When were the studies published, and in what scientific format (e.g., peer-reviewed manuscript, conference proceedings)? Who are the main research teams working on this topic?
2. *Data collection* Which IL research designs (e.g., interval-, signal-, event-, device-contingent design) are used to collect data? Which technology (e.g., paper-and-pencil, smartphone, computer) is adopted to collect data?
3. *Definition and operationalization* How is financial well-being defined when it is investigated at the daily level? Which side (objective vs. subjective) of the construct is investigated? How is it operationalized?
4. *Research questions* Which kind of research questions have been posed in research investigating financial well-being using IL methods?
5. *Data analysis* How have IL data been analyzed? Which software is commonly used?

Responses to those questions are reported in the results section. Based on these results, in the discussion section we provided recommendations for future investigations in the field.

### Searching for Relevant Studies

The second stage of a scoping review is identifying the relevant studies and developing a decision plan for where to search, which terms to use, which sources are to be searched, time span, and language (Levac et al., 2010). We decided to search in three electronic databases: Scopus, PsycINFO, and Econpapers. These databases were chosen as they cover more than 20 disciplines, including those usually interested in the study of financial well-being (Sorgente & Lanz, 2017).

We used the following syntax: ("financial well*" OR "economic well*" OR "financial satisfaction" OR “financial stress” OR "income" OR “money” OR “expense” OR “debt”) AND ("intensive longitudinal" OR "experience sampling" OR "daily diary" OR "interaction records" OR "ecological momentary assessment" OR "ambulatory assessment" OR "real-data capture"). The first parenthesis contained the main expressions used to label objective and subjective financial well-being (Sorgente & Lanz, 2017), whereas the second parenthesis contained the main expressions used to label the IL methods (Bolger & Laurenceau, 2013). The search was performed on January 16th, 2020, with no restrictions to the date of publication or language.

The selection of records was done using two inclusion criteria and two exclusion criteria. The first inclusion criterion consisted of selecting studies in which objective or subjective financial well-being (even if labeled/operationalized as financial stress) was a variable measured in the study. In accordance with the literature presented in the introduction, we considered measures assessing any subjective positive (financial well-being) or negative (financial stress) evaluation of one’s present or future financial condition as an adequate measure of “subjective financial well-being”. We considered a measure of “objective financial well-being” as any information about economic resources (money, assets) that were received/earned (financial well-being) or lost/spent (financial stress). Finally, we also included studies which collected information about financial events that could support (e.g., receiving a job promotion) or stress (e.g., losing the job) one’s financial condition. The second inclusion criterion was the adoption of an IL design, verifying that the repeated measurements were actually longitudinal (at least 5 measurements; Bolger & Laurenceau, 2013) and intensive (at least once a day; Cotter & Silvia, 2019).

Once we selected records satisfying these two inclusion criteria, exclusion criteria were checked. The first criterion was excluding studies in which the variable measuring financial well-being was not collected intensively. Many studies (e.g., Bayraktaroglu et al., 2019) measured income (objective financial well-being) only at the initial assessment, while the intensive measurement concerned variables other than financial well-being. For example, Bayraktaroglu et al. (2019) verified if daily time spent watching TV was related to the individual’s self-reported positive affect, controlling for demographic variables such as income. The second exclusion criterion consisted of removing studies in which the money that was earned/spent or evaluated was not real. In some studies, intensive measurements concerned simulated money movement, like non-real money allocation in a dictator game (e.g., Uziel et al., 2020).

Two researchers independently screened the records obtained from the databases to determine if they met inclusion and exclusion criteria. First, the abstracts were screened. When the abstract information was not sufficient to determine the record’s eligibility, the full-text was examined.

### Selecting Studies

**Fig. 1:**
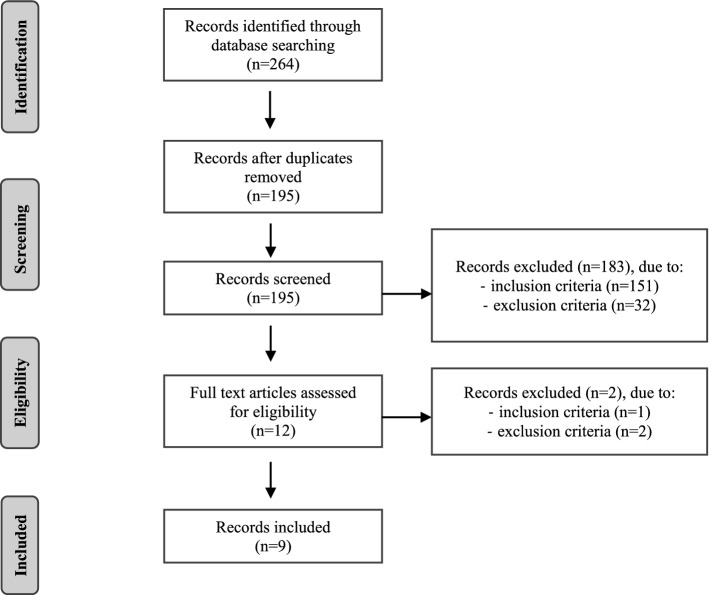
PRISMA diagram of selection process

The third stage of a scoping review is selecting, among the records obtained from the database search, the ones that respect inclusion and exclusion criteria (Levac et al., 2010). In our study, a total of 264 records was obtained from the databases search (133 from Scopus, 92 from PsycINFO, and 39 from Econpapers). After duplicates were removed, 195 records were retrieved for eligibility assessment. Details about the assessment of each of these 195 documents can be found here: https://doi.org/10.6084/m9.figshare.13365245. Nine records met all the eligibility criteria, which is a small number but consistent with the number of studies included in other reviews related to similar topics (e.g., Ghazali et al., 2020; Wilmarth, 2020 included 13 studies each). Further, because our scoping review focuses on the emergence of a new methodology, and our goal is to demonstrate applications and help delineate future research questions to move this small literature forward, we found this number sufficient for this goal. One of the nine records we selected (Joyce et al., 2019) described two different studies. We coded only the second study they presented as the first study did not satisfy inclusion criteria. In Fig. 1 the selection flow is documented in a Preferred Reporting Items for Systematic reviews and Meta-Analyses (PRISMA; Peters et al., 2015).

The last two stages of the scoping review (charting the data, and collating, summarizing, and reporting the results) were realized separately for the five research questions of the current scoping review: diffusion, data collection, definition and operationalization, research questions, and data analysis. For each research question, we extracted information from each record and organized it in a data-charting form (fourth stage of a scoping review; Levac et al., 2010), as reported in the tables of the results section. Finally, for each research question, we describe extracted information to produce an overview of the breadth of the literature (fifth stage of a scoping review; Levac et al., 2010).

## Results

From each of the nine selected records, we extracted information about (1) research field diffusion; (2) data collection methods, (3) financial well-being’s definition and operationalization, (4) research questions addressed, and (5) data analysis. Information referred to each of these five issues is reported in Tables 1, 2, 3 and 4 and described in the following five paragraphs.

**Table 1:** Information about publication and research team

	Year	Format	Journal	Country	Department
Stephens (2003)	2003	Article	American Economic Review	USA	Public Policy and Management
Rio and Zautra (2011)	2011	Article	Health Psychology	USA	Medicine
Sturgeon et al. (2014)	2014	Article	Psychology and Aging	USA	Psychology
Goldstein et al. (2014)	2014	Article	Psychology of Addictive Behaviors	Canada	Applied Psychology and Human Development
Goldstein et al. (2016)	2016	Article	Journal of Behavioral Addictions	Canada	Applied Psychology and Human Development
Torres and Santiago (2018)	2018	Article	Culture diversity and Ethnic Minority Psychology	USA	Psychology
Totenhagen et al. (2018)	2018	Article	Journal of Family Psychology	USA	Human Development and Family Studies
Hing et al. (2019)	2019	Article	Journal of Gambling Issues	Australia	School of Health, Medical & Applied Sciences
Joyce et al. (2019)	2019	Article	Journal of Behavioral Addictions	Canada	Psychiatry

**Table 2:** Research design and data collection

	Design^a^	Intensive assessment		General assessment		Device	Reward	Sample		
		Days	When	Initial	Final			Unit	Size	Age
Stephens (2003)	1	14	Once a day	Yes	NR	Paper and pencil	NR	Consumer unit	9942	NR
Rio and Zautra (2011)	1	30	Once a day	Yes	NR	Laptop computer	Yes	Individual	250	37–73
Sturgeon et al. (2014)	1	30	Once a day	Yes	NR	Tablet computer	NR	Individual	182	40–65
Goldstein et al. (2014)	2	30	3 times per day	Yes	yes	Palm Pilot	Yes	Individual	108	19–24
Goldstein et al. (2016)	2	30	3 times per day	Yes	NR	Palm Pilot	Yes	Individual	108	19–24
Torres and Santiago (2018)	1	7	Once a day	Yes	NR	Paper and pencil	Yes	Individual	58	M = 13.31
Totenhagen et al. (2018)	1	14	Once a day	Yes	NR	Online survey	NR	Dyadic (couple)	100	over 19
Hing et al. (2019)	1	15	Once a day	Yes	NR	Smartphone	Yes	Individual	722	18–84
Joyce et al. (2019)	1	32	Once a day	Yes	NR	Online survey	Yes	Individual	20	M = 30.7

NR = information not reported in the article

^a^Research design was classified according to Bolger and Laurenceau’s (2013) classification, where 1 = interval-contingent design and 2 = signal-contingent design

**Table 3:** Intensive assessment of financial well-being: definition and operationalization

	Construct			Operationalization		
	Variable	Side	Framework	Sub-scale of	Items	Response scale
Stephens (2003)	Daily expenditures	Objective	Stress	No	ad hoc single item for each expense’s category	(dollar)
Rio and Zautra (2011)	Daily financial worry	Subjective	Stress	No	ah hoc single item	4-point scale
Sturgeon et al. (2014)	Financial stressor events	Events	Stress	No	seven ad hoc items	dichotomous
Goldstein et al. (2014)	Money spent	Objective	Stress	Yes, gambling behavior	One item adapted from the GTLFB^a^	(dollar)
Goldstein et al. (2016)	Money spent	Objective	Stress	Yes, gambling behavior	One item adapted from the GTLFB^a^	(dollar)
	Money win		Well-being		One item adapted from the GTLFB^a^	(dollar)
Torres and Santiago (2018)	Daily economic stress	Events	Stress	Yes, daily stress	Five items adapted from MESA^b^	4-point scale
Totenhagen et al. (2018)	Financial satisfaction	Subjective	Well-being	No	ah hoc single item	5-point scale
	Financial stress		Stress		ah hoc single item	5-point scale
Hing et al. (2019)	Money spent	Objective	Stress	No	ah hoc single item	(dollar)
Joyce et al. (2019)	Money spent	Objective	Stress	Yes, gambling behavior	One item adapted from the GTLFB ^a^	(dollar)

^a^Gambling timeline followback (GTLFB; Weinstock et al., 2004)

^b^Multicultural Events Schedule for Adolescents (MESA; Gonzales, Gunnoe, Jackson, and Samaniego, 1995; Gonzales et al., 2001)

**Table 4:** Research questions and data analysis

	Questions^a^	Performed analysis	Software	Rules^b^	Power analysis
Stephens (2003)	2	Linear regression	NR	No	NR
Rio and Zautra (2011)	3	Multilevel analysis	SAS	Yes	NR
Sturgeon et al. (2014)	3	Multilevel analysis	SAS	Yes	NR
Goldstein et al. (2014)	3	Multilevel analysis	HLM	Yes	NR
Goldstein et al. (2016)	1	*t*-tests, chi-square, multivariate logistic regression	SPSS	No	NR
Torres and Santiago (2018)	1, 3	Multilevel analysis, multiple regression	HLM	Yes	NR
Totenhagen et al. (2018)	1, 3	Multilevel analysis	SAS	Yes	NR
Hing et al. (2019)	1	Linear mixed effect model and regular linear regression	R	No	NR
Joyce et al. (2019)	1	Non parametric Wilcoxon rank tests	SPSS	No	NR

NR = information not reported in the article

^a^Research questions typology proposed by Bolger et al. (2003): 1 = aggregate over time, 2 = model the time course; 3 = model within-person process

^b^Evaluating if the record has respected the five rules that should be respected when analyzing intensive data according toBolger and Laurenceau (2013)

### Diffusion

From the nine selected records, we extracted information to infer when (in which years), how (in which scientific format), and where (in which countries and disciplines) the study of daily financial well-being is mainly spread (see Table 1). We found that these studies have been published mainly in recent years. Specifically, 77.78% (7 out of 9) of the studies have been published in the last six years, suggesting that scholars who study financial well-being have only recently applied IL methods. Thus, there is a potential for increased use of these methodologies as they become more widely read and understood.

These records have all been published in an article format (we did not find any thesis, chapter, or other report formats which satisfied the inclusion/exclusion criteria). Finally, the selected articles have been published in different journals except for Goldstein et al. (2016) and Joyce et al. (2019), which were both published in the Journal of Behavioral Addictions, suggesting that financial well-being has been studied within an IL framework more often in relation to addictive behaviors, such as gambling and betting.

Regarding the research team (i.e., list of authors), we verified in which country authors worked and with which department the first author was affiliated. Researchers were located in US (55.56%), Canada (33.33%), and Australia (11.11%), and their studies were mainly in departments of Psychology (55.56%) and Medicine (33.33%). We found only one department (Public Policy and Management) more related to the Economics discipline. Note that for two records (Goldstein et al., 2014, 2016) the research team was the same.

### Data Collection

The research design adopted by each study to collect data was classified using the aforementioned typology by Bolger and Laurenceau (2013): interval-, signal-, event-, and device-contingent design. Most of the reviewed articles (77.78%) adopted an interval-contingent design, asking participants to complete the questionnaire in predetermined moments, while the remaining two studies (Goldstein et al., 2014, 2016) adopted a signal-contingent design, sending random signals to the participants (see Table 2). The interval-contingent studies were daily diary studies as they required participants to complete a survey once a day (often in the evening/night), each day for a consecutive period ranging from 7 days (Torres & Santiago, 2018) to 32 days (Joyce et al., 2019). The only exception was Hing et al., (2019), who collected data for 15 days distributed in three non-consecutive weeks (five days per week). While studies adopting an interval-contingent design required only one assessment per day, the studies adopting the signal-contingent design required participants to report three times per day.

We found that included records did not collect data only intensively. They also collected data before and/or after the intensive period of assessment using a traditional methodology. All articles reported collecting general information from participants before the intensive data collection (i.e., initial assessment or baseline), while only one study (Goldstein et al., 2014) reported collecting data also at the end of the intensive assessment. We surmise this is true also for Goldstein et al. (2016) as these two studies referred to the same data collection, but no information about the final assessment was reported by Goldstein et al. (2016).

The devices adopted to collect data were different across studies. Some researchers adopted paper-and-pencil (22.22%), whereas others required participants to use computer (22.22%) or Palm Pilot (22.22%). One study required participants to use their own smartphone, while the remaining two studies (22.22%) did not specify the device adopted and stated that data were collected through online surveys. More than half of the studies (66.66%) reported rewarding participants for their participation; these rewards were proportional to the number of repeated measures in which the participant took part.

Specific to the sample on which data were collected, three kinds of information were extracted from the articles: unit of analysis, sample size, and participants’ age. The unit of analysis of most studies (77.78%) was the single individual, while the remaining two studies adopted as unit of analysis the consumer unit (household) and the dyad (married and unmarried heterosexual couples), respectively. Sample sizes ranged from a minimum of 20 units to a maximum of 9942. The participants’ ages were very heterogeneous across studies, from studies focusing on adolescents (Torres & Santiago, 2018) to studies including adults across the lifespan (i.e., ages 18–84; Hing et al., 2019).

### Financial Well-Being’s Definition and Operationalization

We dedicated an in-depth analysis to the way in which the financial well-being construct was defined and operationalized for the intensive assessment in the selected articles (see Table 3). We found more than half of the studies (5 out of 9) focused on the objective side of financial well-being, measuring the amount of money spent or earned by the subject, asking participants to report this amount in dollars. The amount of spent money in a variety of categories was examined in only one study (e.g., food, instant consumption, etc.; Stephens, 2003), whereas in the other four studies the amount of (spent or earned) money was specific for a unique category, like gambling (Goldstein et al., 2014, 2016; Joyce et al., 2019) or betting (Hing et al., 2019). In two articles (Rio and Zautra, 2011; Totenhagen et al., 2018), the authors focused on the subjective side of the construct (i.e., subjective evaluation of one’s own financial condition). Finally, the remaining two studies operationalized financial well-being as financial events that have the potential to change the individual’s level of financial stress, such as “ran out of money to cover living expenses” (Sturgeon et al., 2014) or “you could not buy yourself something important because your family could not afford it” (Torres & Santiago, 2018), for which participants indicated if they had experienced each event (yes/no; Sturgeon et al., 2014) or how much they were stressed by each event (Torres and Santiago, 2018).

These two studies were also the only two studies in which the instrument used to intensively assess the construct was a multi-item scale; often single-item scales are adopted in IL studies to reduce participant burden. Items were developed ad hoc (5 records) or adapted from scales that are typically used for non-IL studies (4 records), demonstrating the lack of validated instruments for the intensive assessment of financial well-being.

In most of the cases (7 studies) authors adopted a “financial stress” conceptualization to define financial well-being (e.g., “day-to-day stress associated with residing in poverty”; Torres and Santiago, 2018, p. 210), while the remaining two studies adopted both the “financial well-being” and the “financial stress” conceptualizations. Specifically, Goldstein et al. (2016) measured both the money lost (stress) and won (well-being) through daily gambling behavior, whereas Totenhagen et al. (2018) assessed both the daily perception of financial stress (stress) and financial satisfaction (well-being).

It is important to specify that in almost half of the studies (four out of nine), the financial well-being construct was not the main variable of the study, but was included only as sub-dimension of a more general construct (e.g., financial stress as one type of a variety of daily stress that composed the daily stress scale; Torres & Santiago, 2018). In the other three cases (Goldstein et al., 2014, 2016; Joyce et al., 2019), information about the money spent/earned was collected as an indicator of the gambling behavior.

### Research Questions about Daily Financial Well-Being

Bolger et al. (2003) stated that the effectiveness of IL designs depends on careful consideration of the question(s) one seeks to answer and, consequently, systematized the types of research questions in a 3-type classification: (1) what is the typical person like, and how much do people differ from each other?; (2) how does a typical person change over time, and how do people differ in change over time?; (3) what is the within-person process for the typical person, and how do people differ in these processes?

The first type of research question has a descriptive purpose and can be realized by aggregating the score of the same individual over time. The aggregation can be done by averaging the repeated measures (to obtain a more reliable measurement of the “level” of the construct) or calculating the variability of these measures (to obtain an estimation of the “stability” of this construct across the measurements) to measure “what is the typical person like”. Finally, researchers can investigate which factors (e.g., gender, age, personality trait) are associated with these descriptive scores of “level” and “stability” (see Greenier et al., 1999 for an example of these descriptive scores’ use), explaining “how do people differ from each other”.

Among the nine studies we reviewed, five studies (see Table 4) answered research questions that fit in the first type. For example, Goldstein et al. (2016) assessed how much money young adult gamblers spent and won over 30 days, averaging 90 repeated measures to identify the overall level of won/spent money for individuals. They then tested between-person factors (e.g., online vs. non-online gamblers) associated with differences in levels of won/spent money. This first type of research question does not take into account the temporal dynamic of the construct (i.e., if the time of the day/week in which data are collected affects the response). A response given during the morning counts the same as a response given during the evening, as well as a response given during the weekend counts the same as a response given during the weekdays, etc.

The second type of research question proposed by Bolger et al. (2003) examines the temporal dynamics of a construct. Among the nine studies we reviewed, only one study answered this kind of research question. Stephens (2003) modeled the time course of the expenses that each household (composed of retired and disabled workers along with their dependents and survivors) incurred since the arrival of the Social Security check (i.e., benefit that the United States Social Security Administration gave monthly). The financial well-being variable was operationalized as the amount of money spent each day. Results suggested that the expenditure increased “on the day of the check arrival [“how does a typical person change over time”] and is concentrated amongst households for whom Social Security is the primary source of income [“how people differ in change over time”]” (Stephens, 2003, p. 419).

Finally, the third kind of research question aims to determine the antecedents, correlates, and consequences of daily experiences. Among the reviewed studies, this was the most common research question, found in five different studies (see Table 4). For example, Rios and Zautra (2011) investigated the daily relationship between financial worries and pain severity in a sample of women with osteoarthritis and fibromyalgia, finding that higher levels of financial worries caused greater perception of physical pain on the same day [“what is the within-person process for the typical person”]. Furthermore, they found that this relationship was moderated by women’s general level of economic hardship [“how do people differ in these processes”]. In particular, the relationship between daily financial worries and pain was stronger for women who had a more disadvantaged economic condition.

It is important to specify that some studies addressed more than one kind of research question. For example, Totenhagen et al. (2018) answered two different kinds of research questions, fitting both the first and the third type of Bolger et al. (2003) classification. They estimated the level of stability that individuals had in their daily perceptions of financial satisfaction and financial stress, finding that women reported lower stability than men in their daily financial satisfaction level (first type of research question). Furthermore, they verified if daily financial satisfaction and stress were associated with daily relationship satisfaction (third type of research question). They found that higher daily financial satisfaction was associated with higher daily relationship satisfaction for unmarried women, whereas higher financial satisfaction was associated with lower relationship satisfaction for unmarried men.

### Data Analysis

The last group of information we extracted from the nine selected records concerned data analysis. We verified which kind of analysis was performed, using which software, and if the way in which analyses were performed respected the five rules that Bolger and Laurenceau (2013) proposed for the analysis of IL data: (1) carefully distinguish the between-subjects and the within-subjects level of analysis in the statistical method; (2) allow for random effects, that is, to allow subjects to differ from one another in within-subject processes; (3) take into account the influence of time in the statistical model; (4) specify the appropriate number of independent units (i.e., consider the subject as unit of analysis and not the measurement); (5) choose interpretable zero points for within-subject independent variables (i.e., centering variables).

We found (see Table 4) that most of the studies (five out of nine) performed multilevel analysis and, consequently, respected the guidelines proposed by Bolger and Laurenceau (2013). Multilevel analysis, indeed, has been defined as the most used and useful method to analyze intensive data (Lischetzke, 2014) as it allows researchers to take into account that measurement occasions (Level 1) are nested within subjects (Level 2). Other studies investigated the relationship between variables mainly adopting linear regression, while non-parametric analysis was adopted when the sample size was particularly small (Joyce et al., 2019). The most commonly used software to perform multilevel analysis was SAS PROC MIXED, followed by HLM software. Non-multilevel analyses were instead performed using SPSS or R software.

Finally, we verified if the authors performed a power analysis, as recent publications (e.g., Bolger et al., 2012) recommended researchers determine the number of subjects and time points needed to adequately test key hypotheses for IL study. None of the studies reported any power analysis. This pattern may be because power analysis is more feasible when there are prior data available on which to base the necessary assumptions (Bolger & Laurenceau, 2013). Because IL methods are relatively new and most measures are not yet validated for intensive use, and because non-independence in the data must also be accounted for, guidelines for power analysis in IL research are not yet standard.

## Discussion

By performing a scoping review, we identified and reviewed nine articles which studied financial well-being adopting an IL approach. In these studies, participants belonging to samples different for unit of analysis (individual, dyad, groups) and size (from 20 to over 9000) were asked to assess their financial well-being once per day (interval-contingent design) or more times per day (signal-contingent design). Across these nine studies, both sides (objective and subjective) of financial well-being were investigated: daily objective financial well-being was operationalized as money spent (stress) or earned (well-being) during the day, whereas daily subjective financial well-being was operationalized as the level of financial stress or financial satisfaction perceived during the day. In general, the stress conceptualization was more often used than the well-being one, perhaps because the construct of stress is investigated using an IL approach more than well-being is (e.g., Google Scholars’ records that have the expression “daily stress” and “daily well-being” in their title are 1,060 and 166 respectively). These studies investigated different types of research questions showing that (1) financial well-being changes daily, (2) its change over time can be modeled, and (3) it is possible to identify within-subject processes that explain this change. Analysis performed to answer these questions often consisted of multilevel analysis. This review of literature was useful to identify the main gaps of this research field and suggest future research directions.

### Future Directions for the Study of Financial Well-Being Using an IL Approach

Although the scoping review methodology specifies that the review should generate a future research agenda (Arksey & O’Malley, 2005), specific guidelines are not provided about how to organize this agenda. Here, we outline recommendations for future research on daily financial well-being specific to theory, contexts, and methodology following the recommendations of Paul et al. (2017).

#### Future Directions: Theory

Around half of the studies included in this review assessed financial well-being as a sub-dimension of other constructs (e.g., daily stress, gambling behavior), demonstrating a lack of theoretical reflection on daily financial well-being itself. We suggest future researchers seek to clarify (1) daily financial well-being’s definition and theory of change; (2) the relationship between components of daily financial well-being constructs; and (3) the relationship between daily financial well-being and other constructs.

Although numerous studies (Brüggen et al., 2017; Joo, 2008; Sorgente & Lanz, 2017; Wilmarth, 2020) have provided a clear definition of financial well-being as a positive financial condition that has an objective (possessed material resources) and a subjective side (perception of one’s present and future financial condition), none of the included studies referred to those definitions to develop an adaptation of such definition at the daily level. We argue that, at daily level too, financial well-being should be investigated taking into account both its objective (daily earnings or expenses) and subjective sides (daily perception of one’s financial condition). Instead, none of the included studies recognized the multidimensionality of the construct, investigating only one side of financial well-being. We believe that a more comprehensive view of daily financial well-being could be born from the joint work of economists and psychologists and could produce a widely recognized definition of daily financial well-being.

A reflection on daily financial well-being’s definition can be helpful to also develop a theory of change for this construct. “For example, if researchers (1) theorize that a given physiological variable fluctuates every hour, (2) data must be collected at least on an hourly basis” (Windt et al., 2018, p. 2). Consequently, it is important to have a clear definition of the construct of interest before performing an IL research design in order to decide the correct time interval between measurements. None of the included papers explicitly stated how often they expect that financial well-being changes, but we found that most of the studies collected the financial well-being variable once per day. This suggests that most of the researchers have the theoretical assumption that financial well-being is a construct that changes daily, but we also found studies (e.g., Goldstein et al., 2014, 2016) that expected to find variations of this construct within the same day (three measurements per day). We presume these assumptions are implicitly related to which side (objective or subjective) of the construct authors were interested in. It seems that when their focus was on the cognitive evaluation of the financial condition (e.g., financial satisfaction), researchers expected that financial well-being does not change within the same day and only measured it once per day (e.g., Totenhagen et al., 2018), whereas when researchers focused on the objective side of the construct (e.g., money spent gambling), they expected to find differences across the day and collected multiple assessments per day (e.g., Goldstein et al., 2016). Yet, these assumptions were not explicitly stated nor clearly connected to theory. It is important to stress that these studies expecting more changes in the same day tended to focus on potentially problematic uses of money (e.g., gambling). It may be that, outside of this addictive behavior framework, it is sufficient to use once-daily intervals to sample financial well-being change both for the subjective and objective side, but future work should continue to clearly state and/or develop theoretical assumptions about timelines for changes.

Another relevant theoretical improvement that future research should realize is the study of the relationships that the different components of daily financial well-being (e.g., objective and subjective, present and future) have with each other. Classic cross-sectional and longitudinal studies have demonstrated that objective financial well-being tends to predict the subjective side (e.g., Shim et al., 2009) and that there is a positive relationship between the perception of the present and future financial conditions (e.g., Iannello et al., 2020), yet it is unclear if these same patterns hold with respect to their daily associations. Empirical investigations of these links using IL methods (e.g., daily diaries) would help to support and/or refine theoretical frameworks that have been built and tested using more classic designs. Furthermore, this evidence could help financial policy makers to redesign their financial interventions and policy. In particular, financial policy makers tend to focus on objective assessments of financial well-being/stress (e.g., income, debt service to income ratios), giving much less attention to the subjective side of the construct (Brzozowski & Visano, 2020). IL methods could help in providing evidence of whether a within-person change in subjective financial well-being/stress can promote financial behaviors that will, in turn, modify the individual’s objective financial condition.

Finally, despite not all the scientific community agreeing about financial stress as a component of financial well-being, we argue that future IL studies should measure both financial aspects (well-being and stress) to investigate the daily relationship occurring between them. Using IL methods would help to disentangle if these two concepts are two sides of the same construct or rather related but distinct constructs by permitting investigation of how each construct fluctuates or changes, and whether and how those changes in stress and well-being occur together or whether a change in one precipitates a change in the other. These investigations will help to clarify and refine theory on how financial stress and well-being are intertwined. In particular, examining within-person consistency across various operationalizations of financial well-being and financial stress will help to (1) elucidate which are the sub-dimensions of each of the two concepts; (2) clarify if these two concepts are just two sides of the same construct; and, consequently, (3) formulate a more precise definition of financial well-being/stress.

The last theoretical aspect important for the future research agenda is the study of the relationship that financial well-being has with other constructs. Classic cross-sectional and longitudinal studies (e.g., Iannello et al., 2020; Shim et al., 2009, 2010) have outlined a nomological network of financial well-being. In particular, it is widely recognized that both the objective and subjective (both present and future perception) sides of financial well-being are predicted by both individual (e.g., financial behavior, financial capabilities, etc.) and contextual factors (e.g., economic growth rate, consumer protection, etc.) and that, in turn, financial well-being affects general well-being (psychological well-being, mental health, quality of life). This knowledge has been developed performing between-subject comparisons. Using IL data, future studies could verify if this nomological network holds at within-level too. In fact, the same variables can have different relationships at between- and within-level (Lischetzke, 2014). For example, we can imagine that in the day in which one performs a healthy financial behavior (e.g., save money instead of buy new shoes), one’s subjective financial well-being perception will decrease (“I do not have enough money to buy what I desire”), so in a within-subject evaluation financial behavior and well-being could be *negatively* related. Alternatively, asking one to evaluate in general how often he/she performs healthy financial behaviors and his/her overall financial well-being on just one occasion may yield different results. We may find a *positive* relationship between the two constructs as one who often behaves well (e.g., regularly saves money) may perceive his/her financial well-being to be high (e.g., has a lot of money in the bank and perceives high financial satisfaction). In sum, by empirically testing theoretical assumptions built from classic research designs using IL methods, these theoretical assumptions can be more rigorously tested and theoretical frameworks can be refined to be more accurate and nuanced.

#### Future Directions: Context

The current review showed that half of the records were published in journals specialized on addictive behavior. This means that daily financial well-being has thus far been mainly investigated with respect to populations characterized by behavioral (e.g., gambling or betting) disorders, offering limited knowledge about the general population. Future studies should examine more generalizable samples as we can expect that individuals not affected by addictive disorders can also have financial behaviors and perceptions that change daily (e.g., Sturgeon et al., 2014; Totenhagen et al., 2018). Future studies should also consider gender and the age of their sample. Regarding gender, previous studies (Totenhagen et al., 2018) have verified that daily financial well-being can present different levels of variability between women and men, suggesting that causes of this variation may be different across genders. Future studies should collect data from both men and women and results obtained should be compared across these two groups. Regarding age, we suggest examining a population that is homogeneous for the stage of life that participants are living. As suggested by Salignac et al. (2019) “financial well-being must be understood within a life-course framework—this includes the stage people are at in the life-course (early childhood, childhood, adolescence, young adulthood, adulthood, older adulthood) and major events that have an expected or unexpected financial shock (e.g. birth of a child, death of a loved one, relationship breakdown, need to move homes, unemployment etc.)” (p. 6).

Finally, future IL studies should examine populations in contexts other than the US, Canada and Australia. This review verified that daily financial well-being has been investigated using IL methods only in these three countries, all of which are in the top 15 richest countries in the world (The World Bank, 2018). The experience of daily financial well-being in the richest countries likely does not represent well what is going on in the rest of the world, as it has been demonstrated that both objective and subjective financial well-being are affected by the contexts in which individuals live (e.g., Sunal et al., 2013). Future studies should try to collect data also in less advantaged countries. Ideal would be the realization of cross-cultural studies that could verify the impact that the country has on the experience of financial well-being.

#### Future Direction: Methodology

In this section we highlight three important methodological considerations for future research: (1) data collection, (2) measurement of variables, and (3) data analysis. Iida et al. (2012) indicated that in IL studies the data collection can happen through three different formats: paper-and-pencil format, brief telephone interview, and electronic response format. Our review found only one study (the oldest one; Stephens, 2003) using the paper-and-pencil format, whereas all the others collected data in electronic format. Furthermore, within studies using the electronic format, we found some differentiations: while the oldest studies provided participants the device to use to fill in the survey (e.g., Palm Pilots), the most recent studies did not. Instead, these studies tended to send an online link that participants could use from any own device (e.g., smartphone, tablet, computer). Although it is now quite common for people to have access to electronic devices, it is by no means ubiquitous. For example, according to the Federal Communications Commission, about 6% of the US population lacks access to reliable internet, and this rate can approach 25% in rural areas. Therefore, it is critical for researchers to consider the methodology being used in light of generalizability of the people able (or willing) to complete the research using that methodology. Further, researchers may need to consider participant reward structures that incentivize completion of these intensive, repeated surveys. Compared to traditional studies, IL studies can be much more time-consuming for participants, so researchers might consider rewards that are proportional to the number of repeated measures in which the participant took part (e.g., compensation per survey completed, bonuses for meeting thresholds, etc.).

Another methodological aspect that requires researchers’ attention is the measurement of daily financial well-being. As reported in Table 3, reviewed studies adopted items that were developed ad hoc or adapted from scales that are usually used for non-IL studies, generating measures that do not have evidence of validity and that do not allow cross-study comparisons. Future studies should work to design and test instruments that comprehensively measure the different components of daily financial well-being. The psychometrics of such instruments could be tested following the suggestions proposed by Bolger and Laurenceau (2013) that indicated statistical techniques (such as the Multilevel Confirmatory Factor Analysis) useful to collect validity evidence for new instruments developed within an IL framework.

Finally, we suggest researchers carefully consider data analysis in future studies to capture all the variability present in this type of data. Procedures to adopt a descriptive approach to data have been proposed (Greenier et al., 1999; Wright & Zimmermann, 2019), but when researchers are interested in the relationships between variables, the descriptive approach is not sufficient. Although multilevel analysis is a well-recognized solution, we also suggest researchers explore opportunities offered by newly implemented models to study dynamic change (for an overview, see Asparouhov & Muthén, 2020). Finally, we suggest researchers perform power analysis to inform the sample size and number of observations needed for their study. None of the studies included in the current review reported power analysis information, yet there are specialized publications (e.g., Bolger & Laurenceau, 2013; Bolger et al., 2012) that report how to execute it in the IL framework and determine the number of subjects and time points needed to adequately test hypotheses.

All the studies presented in the current review refer to quantitative IL data, and indeed it is rare to find examples of studies using qualitative methods along with IL methods. Still, it is possible to use IL methods to collect qualitative data and doing so may provide more nuance in people’s lived experiences than quantitative methods can access. For example, Vleioras et al. (2008) asked participants daily to report their experiences and emotions by writing on diary sheets for a period of five months in order to study how adolescents develop a mature view of themselves. Although this example is not specific to financial well-being, it demonstrates the implementation of IL methods to collect qualitative data that may provide more insight into participants’ thoughts, feelings, and reflections, which may be helpful for understanding participants’ daily emotions surrounding their financial well-being.

### Limitations

Although we aimed to stress the opportunity that IL approaches can offer to the study of financial well-being, we also recognize that this approach has some disadvantages including (1) increased cost in term of data management, (2) complexity of data analysis, (3) increased management of technology for data collection, (4) higher intrusiveness perceived from participants, (5) time-consuming for participants, (6) risk of reactivity as participating in a study may itself be an intervention that influences rating of experiences, and (7) retrospective biases that, although reduced, may still be present to some degree, for example when at the end of the day a participant is asked to recall how the morning was (e.g., Bolger & Laurenceau, 2013; Bolger et al., 2003).

We recognize that our review has some limitations. The main one is related to the small number of included records. Although we performed a systematic search in three comprehensive databases (Scopus, PsycINFO, Econpapers), we found only nine records respecting the inclusion and exclusion criteria. Thus, the current review can be seen as an early sampling of the literature to help shape where it continues to move forward. Given the increased rate of publications examining financial well-being using IL methods in recent years, we expect a continued increase of studies investigating financial well-being using these methodologies in the following years. Use of these methods are particularly relevant in the current context. Considering the economic impact that the COVID-19 pandemic will have in many countries, the adoption of IL methods to study objective and subjective well-being in these turbulent times would add rich and nuanced evidence about how financial well-being unfolds, fluctuates, and dynamically interacts with individual and family well-being.

Finally, we acknowledge that because we used systematic (scoping) review methodology, there may be other research that is related in content but does not meet inclusion criteria. For example, there are two books which investigate how families across the world manage their money on a daily basis (Collins et al., 2009; Morduch & Schneider, 2017), but because they did not collect data on a daily or “intensive” basis, they were not included in the present review. However, we felt it important to focus on research examining financial well-being using IL methods specifically, rather than studies examining daily finances using other non-intensive methodologies.

## Conclusion

The current study systematically reviewed nine articles which adopted IL approaches to study financial well-being. Results of this review suggest that in the study of financial well-being, the use of IL methods is still unfolding. The temporal trends of previous publications (e.g., the increased number of publications using IL methods in recent years) suggest that we may see a continued increase in utilization of these research designs. Through this paper we aimed to show the opportunity that IL designs can offer to the financial well-being literature and suggest future research directions, but it is important to specify that we do not suggest that all studies should use only the IL approach. IL designs have some limitations and are not always useful; for example they are not adequate to study rare events (e.g., winning the lottery or receiving a change in salary). As Bolger and Laurenceau (2013) clarified, an adequate understanding of human behavior requires both traditional and IL approaches. We suggest to researchers that IL methods can serve as an important complement, but not complete replacement, to more traditional (e.g., cross-sectional, longer term longitudinal, etc.) methodologies. The use of IL methods opens doors to the examination of new research questions and clarification of within-person processes and mechanisms involving financial well-being that will ultimately serve to move the science forward.

## Data Availability

Data available within the article or its supplementary material.
